# ASBR-CL: Stage-Aware Balanced Replay for Memory-Limited Continual Fault Diagnosis

**DOI:** 10.3390/s26144416

**Published:** 2026-07-11

**Authors:** Jiaxin Li, Guanghe Zhu

**Affiliations:** College of Computer Science and Technology, Xinjiang Normal University, Urumqi 830054, China; 107622025210553@stu.xjnu.edu.cn

**Keywords:** vibration-based sensing, industrial fault diagnosis, continual learning, exemplar memory, balanced replay, catastrophic forgetting

## Abstract

Vibration-based sensing systems for deployed industrial fault diagnosis often face incremental fault classes, changing degradation stages, and limited permission to retain historical sensor streams. Static fault classifiers are therefore insufficient for online maintenance settings in which a model must learn new sensor-observed states while preserving previous diagnostic knowledge under a bounded memory budget. This paper proposes ASBR-CL, an adaptive stage-aware balanced replay framework for continual fault diagnosis under a fixed exemplar-memory budget. ASBR-CL combines dataset-adaptive exemplar memory, balanced replay between current data and retained exemplars, and a conditional validation checkpoint module that is enabled only when it improves balanced stage recognition. Experiments on SEU-enhanced92, the 92-dimensional feature construction for SEU, and XJTU-bearing23, the 23-dimensional feature construction for XJTU-SY, compare ASBR-CL with DGGN/MFF same-backbone continual-learning baselines and XJTU imbalance-aware variants under five random seeds and K=100 training exemplars. On SEU, the selected ASBR-CL setting reports 97.49±1.18 Average Accuracy, 90.37±4.53 Final Accuracy, 90.37±4.53 Macro Recall, and 11.50±5.25 Average Forgetting. On XJTU, the conservative ASBR-CL-BoundedVal-K100 setting is not a universal Final Accuracy winner: DGGN-ER reaches a slightly higher Final Accuracy (89.52±2.68 versus 89.05±2.48). The ASBR-CL evidence instead lies in balanced recognition and retention, with Macro Recall 75.00±2.00, Macro-F1 67.66±3.83, and Average Forgetting 20.39±4.89, compared with DGGN-ER at 63.94±7.50, 55.48±12.82, and 41.11±13.61. Additional validation-resource, RMS-derived stage-definition, and imbalance-aware baseline analyses show that the revised XJTU claim should be framed as more stable Macro Recall, Macro-F1, and forgetting control under memory-limited continual diagnosis, not as superiority on every accuracy metric.

## 1. Introduction

Vibration-based sensing has become a practical route for monitoring rotating machinery, bearings, gearboxes, and other industrial assets because sensor signal streams can be collected continuously and transformed into discriminative health-state representations [1,2]. Most deep fault diagnosis studies, however, still evaluate models in a static setting: the available fault classes and operating conditions are assumed to be known before deployment, and historical samples remain accessible for retraining [3,4]. This assumption is restrictive in industrial sensing deployments where new fault modes, degradation stages, or operating domains appear after the diagnostic model has already been installed on a monitoring pipeline.

Continual learning addresses this deployment gap by updating a model from a sequence of tasks while retaining previously learned knowledge [5,6]. In fault diagnosis, this is not only a machine-learning convenience but also an engineering requirement. If a model adapts to new fault states by overwriting old states, it can create unsafe blind spots; this loss is the central catastrophic-forgetting problem measured in continual-learning benchmarks [7,8]. Conversely, if the model preserves old states by storing all historical vibration windows or by retraining from scratch, the update cost, storage load, and data-governance burden can become incompatible with online diagnostic deployment. A deployed diagnostic system therefore needs an incremental update mechanism that is memory-limited, retention-aware, and sensitive to minority or late degradation stages. Recent continual fault diagnosis studies have started to examine class-added and domain-varying settings for motors and rotating machinery [9,10], but the memory-limited replay setting still requires careful control of old-new sample balance, class imbalance, and model-selection bias.

This paper studies continual fault diagnosis for vibration sensor streams under a fixed exemplar-memory budget, where the learner can retain only a bounded training exemplar memory after each incremental step. The setting differs from offline fault classification and from joint training: the model receives new diagnostic sensor data sequentially, cannot revisit all previous training samples, and must preserve balanced recognition across old and new fault states. We distinguish this training-memory limit from validation and test resources: validation data may be retained separately for model selection in the validation-assisted XJTU protocol, while test data are used only for final reporting. Average or final accuracy alone is not sufficient in this setting. In degradation-stage diagnosis, a model can obtain high overall accuracy while failing on minority or late degradation stages, a risk that is consistent with broader studies on imbalanced-domain evaluation [11]. We therefore evaluate not only Average Accuracy and Final Accuracy, but also Macro Recall and Average Forgetting, which better reflect safety-relevant balanced recognition.

We propose ASBR-CL, an adaptive stage-aware balanced replay framework for memory-limited continual fault diagnosis. The method combines three ideas. First, the exemplar memory is selected adaptively according to the dataset structure: random diversity-based replay is used for the stable SEU setting, while stage-aware memory is used for XJTU degradation-stage streams. Second, balanced replay controls the contribution of old exemplars and current-task data so that new learning does not dominate retained knowledge. Third, validation checkpoint selection is treated as a conditional module rather than a mandatory component. It is enabled for the XJTU degradation-stage protocol, where validation Macro Recall aligns with the balanced-recognition objective, and disabled for the stable SEU class-incremental protocol.

The SEU Mechanical Dataset is a public machinery fault diagnosis dataset widely used for gearbox and bearing diagnostic studies. In this work, it is used to construct a class-incremental single-fault diagnosis stream. The processed SEU-enhanced92 setting denotes the 92-dimensional feature construction used in our continual-learning protocol and represents a relatively stable class-incremental diagnostic scenario. The XJTU-SY Bearing Dataset is a public accelerated run-to-failure rolling bearing dataset. We use it to construct an RMS-derived degradation-stage continual diagnosis stream. The processed XJTU-bearing23 setting denotes the 23-dimensional feature construction for XJTU-SY. Its Normal, Slight, Moderate, and Severe labels are RMS-derived condition stages rather than externally annotated degradation ground truth. Together, these two datasets provide complementary continual-diagnosis settings: SEU evaluates retention under a class-incremental single-fault stream, whereas XJTU-SY evaluates balanced recognition and forgetting under an imbalanced degradation-stage stream.

The experimental interpretation is dataset-specific. On SEU, the selected ASBR-CL setting improves the reported same-backbone metrics under the selected protocol. On XJTU, ASBR-CL is not designed or claimed as an overall-accuracy maximizer; instead, the reviewer-compliant ASBR-CL-BoundedVal-K100 setting targets balanced degradation-stage recognition and retention under a fixed training-exemplar memory budget and a bounded validation resource. The strongest capacity-controlled comparison is against DGGN/MFF same-backbone baselines, including DGGN-Fine-tuning, DGGN-ER, DGGN-iCaRL, and DGGN-LwF. Additional imbalance-aware baselines are used to check whether balanced replay or loss reweighting alone explains the result. The method is therefore positioned as a memory-limited continual diagnosis framework that improves balanced recognition and retention where those metrics matter, rather than as an accuracy-only method.

The contributions are summarized as follows.

1. We formulate memory-limited continual fault diagnosis as a balanced-recognition problem under a fixed training-exemplar budget, where fault classes or RMS-derived condition stages arrive incrementally and retained training exemplars, validation resources, and final test evaluation are explicitly separated.
2. We propose ASBR-CL, a dataset-aware continual diagnosis framework that combines adaptive or stage-aware exemplar memory, balanced replay, and conditional validation checkpoint selection, with different selected variants for SEU and XJTU.
3. We provide a revised evaluation protocol with DGGN/MFF same-backbone baselines, validation-resource ablations, RMS-derived stage-definition sensitivity, imbalance-aware baselines, five-seed statistical tests, memory-budget sensitivity, and measured update cost.
4. We report claim-specific findings: SEU supports strong same-backbone performance, whereas XJTU is treated as a harder degradation-stage case in which ASBR-CL-BoundedVal-K100 improves Macro Recall, Macro-F1, and forgetting control without claiming universal Final Accuracy superiority.

## 2. Related Work

### 2.1. Machinery Fault Diagnosis

Deep models are now common in machinery fault diagnosis. They transform vibration windows into health-state labels with less manual feature engineering than classical feature pipelines. Reviews of machine fault diagnosis and machine health monitoring show the evolution from feature engineering toward deep representation learning and benchmarked diagnostic models [2,3,12]. Open benchmark studies further support reproducible rotating-machinery diagnosis [4,13]. Earlier bearing and rotating-machinery studies established the diagnostic value of vibration analysis for rotating assets [14,15]. Deep CNN-based methods then improved raw-signal and anti-noise diagnosis in industrial settings [16,17].

Public gearbox, bearing, and run-to-failure datasets have supported this progress by enabling reproducible evaluation across static diagnostic protocols. Recent work also extends diagnostic learning to limited-sample and cross-machine settings [18]. Much of this literature still assumes that the training and test label spaces are fixed. The resulting classifiers can be strong in static diagnosis, but the assumption becomes restrictive when new fault states arrive after deployment.

This paper focuses on the continual version of the same engineering problem. The aim is not to replace static diagnostic modeling, but to study how a deployed model can be updated when only a compact exemplar memory is retained. This shift changes the evaluation emphasis: final accuracy remains important, but balanced recognition and forgetting become necessary because a high overall score can hide weak recognition of minority or late degradation stages.

### 2.2. Continual Learning and Catastrophic Forgetting

Continual learning aims to update a model over a sequence of tasks or data distributions without catastrophic forgetting. Earlier studies identified rehearsal and pseudorehearsal as direct mechanisms for reducing forgetting in connectionist networks [7], and modern surveys distinguish task-, domain-, and class-incremental settings while organizing methods around regularization, distillation, replay, dynamic architectures, and exemplar-free adaptation [5,6,19]. These general categories motivate the baseline set used in this paper, but they do not directly resolve the engineering constraints of fault diagnosis. Mechanical fault streams are often class-imbalanced, stage-ordered, and safety-sensitive. A method that maximizes overall accuracy may still under-recognize minority degradation stages. ASBR-CL therefore uses replay but makes the memory and checkpoint behavior conditional on dataset structure and evaluates balanced metrics in addition to accuracy.

### 2.3. Continual and Incremental Fault Diagnosis

Continual learning has recently been introduced into fault diagnosis to avoid repeated retraining when new fault types or operating conditions appear. Early lifelong and residual-network studies examine rotating-machinery diagnosis under domain increments and changing operating conditions [20,21]. Self-driven continual learning for class-added motor diagnosis uses unseen-fault detection and propensity distillation to trigger incremental learning [9]. Knowledge-distillation methods have also been used for class-added motor diagnosis, and KDN extends class-added continual learning to cross-machine diagnosis with limited samples [22,23]. Uncertainty-aware continual learning for rotating machinery addresses class, domain, and mixed continual scenarios with pseudo-labeling and prototypical feedback [10], while dynamic branch layer fusion studies continual rotating-machinery diagnosis from an adaptive network-fusion perspective [24]. These works confirm that continual diagnosis is an active engineering problem, but many methods focus on unseen-fault detection, new-fault adaptation, distillation, pseudo-labeling, or architectural adaptation rather than the memory-limited replay balance studied here.

ASBR-CL targets the complementary setting where a small exemplar memory is available and must be allocated carefully. This setting is close to practical maintenance constraints: retaining a compact memory can be more realistic than keeping all historical windows, while still allowing replay to stabilize old decision boundaries. The key design question becomes which exemplars should be retained, how old and new data should be balanced during updates, and whether validation-based checkpoint selection improves balanced recognition on a given dataset.

### 2.4. Exemplar Replay and Model Selection

Exemplar replay is one of the most direct ways to reduce forgetting when memory is allowed. In diagnostic streams, replay raises two additional questions. First, the retained exemplars should reflect the structure of the fault stream rather than only the majority classes. Second, replay batches should not let current-task samples dominate the small retained memory. Recent continual fault-diagnosis work also shows that limited-sample cross-machine settings need explicit mechanisms for preserving old diagnostic behavior while adapting to new classes [18,23].

Model selection is also non-trivial in continual diagnosis. Selecting a checkpoint by overall validation accuracy may favor majority or easier states, while selecting by a balanced metric can be useful when degradation-stage recognition is the target. ASBR-CL therefore treats validation checkpoint selection as a conditional module. It is enabled with validation Macro Recall on XJTU, but disabled on SEU because the ablation shows that it reduces final-stage balanced performance there.

### 2.5. Bearing Degradation, Datasets, and Evaluation Metrics

SEU and XJTU provide complementary public diagnostic settings. SEU supports a stable class-incremental diagnosis setting where the selected ASBR-CL path focuses on old-class retention without validation checkpointing. XJTU supports a harder degradation-stage setting where class/stage balance is more important than overall accuracy alone. The dataset-specific construction details and source references are given in Section 4, where the diagnostic streams are defined for the continual-learning protocol.

For this reason, the experimental section reports Average Accuracy, Final Accuracy, Macro-F1, Macro Recall, Average Forgetting, and signed BWT together. Macro Recall is emphasized on XJTU because it captures balanced recognition over RMS-derived condition stages, while Average Forgetting captures retention loss after incremental updates. On XJTU, ASBR-CL-BoundedVal-K100 improves Macro Recall, Macro-F1, and forgetting relative to the same-backbone baselines while not being uniformly superior in Final Accuracy.

### 2.6. Balanced and Imbalanced Fault Diagnosis

Fault diagnosis data are frequently imbalanced because normal states or early degradation stages may dominate the data stream, while severe or transitional fault states are rarer. General imbalanced-learning studies show why conventional accuracy can be misleading and why resampling, reweighting, and balanced losses are often needed [11,25,26,27]. Reviews of small and imbalanced diagnostic data argue that this issue is especially important when minority classes are safety-relevant [28]. Recent bearing-diagnosis studies continue to address the same issue with improved convolutional models and imbalance-aware designs [29]. In a continual setting, imbalance interacts with catastrophic forgetting: a model may retain high overall accuracy while losing minority-stage recall or overwriting old states. This motivates the use of Macro Recall and Average Forgetting as main evidence rather than reporting only average accuracy.

The gap addressed here is therefore specific. Existing diagnostic and continual-learning methods often prioritize overall accuracy, new-fault adaptation, or distillation-based forgetting reduction. ASBR-CL instead focuses on memory-limited replay under a fixed exemplar budget, where balanced old-new sampling, dataset-aware memory selection, and conditional checkpointing are evaluated by their effect on balanced recognition and forgetting control.

## 3. Materials and Methods

### 3.1. Problem Formulation

We consider a class- or stage-incremental fault diagnosis stream $S=\{D_{1},\ldots ,D_{T}\}$, where incremental step *t* provides a current training subset $D_{t}={\left\{\left(x_{i},y_{i}\right)\right\}}_{i=1}^{n_{t}}$ and expands the cumulative diagnostic label set to $Y_{1:t}$. The learner updates parameters $\theta_{t}$ sequentially and is evaluated on cumulative test classes or degradation stages after each step. Historical samples are not fully available during every update. Instead, the learner can retain only a bounded exemplar memory $M_{t}$ after step *t*, subject to

(1) $$|M_{t}|\leq K.$$

Here, $M_{t}$ denotes the retained exemplar memory after incremental update *t*, $|M_{t}|$ denotes the number of retained training exemplars, and K=100 in the main experiments. This setting differs from offline joint training because the latter has access to all historical training data and is therefore reported only as an upper bound.

The memory budget *K* counts only training exemplars stored in the replay memory. The validation split is held out from training, is never sampled for replay, is not inserted into the exemplar memory, and is not counted in *K*. At incremental step *t*, validation checkpoint selection, when enabled, uses the cumulative validation set for all observed classes or stages, denoted by $D_{1:t}^{\mathrm{val}}$; the current-stage validation subset is denoted by $D_{t}^{\mathrm{val}}$. This cumulative validation set contains old-task validation data and current-task validation data so that checkpoint selection can evaluate both new-task learning and old-task retention. The test split is used only for final reporting and is not used during epoch-level checkpoint selection.

### 3.2. Validation Resource and Memory Accounting

The memory budget in this paper is an exemplar-memory budget. It counts only retained training samples in the replay memory $M_{t}$. Validation samples are stored separately for model selection and checkpoint selection; they are not replayed, not inserted into exemplar memory, and not used to update model parameters. At incremental step *t*, the original cumulative-validation setting contains validation samples from the seen classes or stages 1:*t*. This validation resource is therefore separate from *K* and should be interpreted as a model-selection resource rather than as training memory.

This accounting also defines the limitation of the memory-limited claim. In stricter deployments, old validation samples may also be storage-constrained or unavailable. Therefore, the original XJTU cumulative-validation result should be interpreted as validation-assisted continual learning under a fixed exemplar-memory budget, not as a protocol where every labeled validation sample is counted in the same storage budget as retained training exemplars. To address this point, the revision reports ASBR-CL-NoCkpt, ASBR-CL-CurrentVal, and ASBR-CL-BoundedVal-K100. The bounded-validation setting keeps at most 100 validation samples in total for checkpoint selection at each incremental update stage and is the conservative XJTU row emphasized in the revised results. Future deployment-facing protocols may further count validation data in a total labeled-storage budget or use fully validation-free checkpoint rules.

### 3.3. ASBR-CL Framework

**Figure 1:**
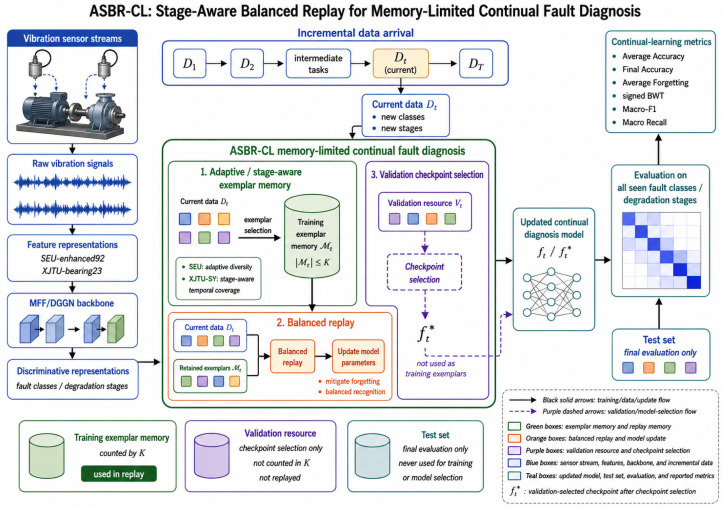
Overall framework of ASBR-CL for memory-limited continual fault diagnosis. The framework separates the vibration-sensor stream, feature construction, exemplar-memory update under budget *K*, balanced replay training, validation checkpoint selection, and final test evaluation. Training exemplars are counted by *K*, validation samples are used only for checkpoint selection, and test samples are reserved for final reporting.

Figure 1 gives the implementation-level flowchart of ASBR-CL. The pipeline separates the training exemplar memory, validation resource, and final test evaluation. This separation is important because only the retained training exemplars are counted in *K*, whereas validation data are used only for checkpoint selection and test data are never used for model selection.

**Figure 2:**
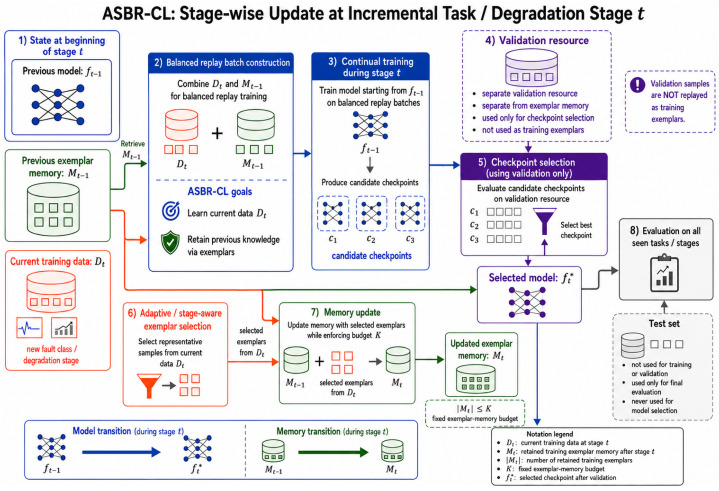
Incrementallearning workflow of ASBR-CL at task or stage *t*. After the current data $D_{t}$ arrive, the model is updated with balanced replay, exemplars are selected, and the retained exemplar memory $M_{t}$ is maintained under the budget *K*. Here, $|M_{t}|$ denotes the number of retained training exemplars, while validation samples are stored separately for checkpoint selection and are not counted in *K* unless a bounded-validation setting is explicitly used.

ASBR-CL is built on the DGGN/MFF continual diagnosis pipeline but changes the memory and update behavior according to the dataset structure. Figure 1 shows the overall implementation framework, Figure 2 gives the incremental update workflow for task or stage *t*, and Algorithm 1 lists the implementation-faithful update procedure. The method has three operational components: adaptive or stage-aware exemplar memory, balanced replay, and conditional validation checkpoint selection.

The design is informed by the broader continual-learning literature: regularization, distillation, exemplar memory, and experience replay address forgetting through different mechanisms [30,31,32,33]. Rebalancing methods in class-incremental learning also motivate explicit attention to old-new sampling bias [34,35]. ASBR-CL does not implement all of these mechanisms as new modules; rather, it uses this baseline landscape to motivate a focused replay-based design for fault diagnosis under a fixed memory budget.
**Algorithm 1.** ASBR-CL continual update
Receive the current incremental training subset and the retained exemplar memory.Select the memory rule from the dataset structure: random-boundary-diversity memory with random coverage for SEU or stage-aware memory for XJTU.Update the bounded exemplar memory under budget *K*.Train with the current subset and old exemplars; if selected, use balanced replay with the configured old-sample ratio.If validation checkpointing is enabled for the dataset, select the checkpoint by validation Macro Recall; otherwise keep the standard final update.Evaluate cumulative diagnosis performance with accuracy, Macro Recall, and forgetting metrics.

### 3.4. Adaptive/Stage-Aware Exemplar Memory

The exemplar memory is selected after each incremental step under the fixed memory budget. ASBR-CL does not use one selection rule for all streams. After step *t*, if $C_{t}$ classes or stages have been observed, the memory is allocated approximately uniformly, with a per-class or per-stage quota of $\left\lceil K/C_{t}\right\rceil$ exemplars. For SEU, the selected configuration uses random-boundary-diversity memory with a 0.10 random coverage ratio. This keeps the diversity-oriented behavior of boundary-diversity selection while adding a small random coverage component. This choice is appropriate for the more stable SEU setting, where the main risk is forgetting old fault classes after new-class updates.

For XJTU, the selected bounded-validation configuration uses stage-aware memory. XJTU-SY source order corresponds to degradation progression because the source files in each bearing run are ordered by sampling time, and each source file corresponds to one-minute interval sampling. Within each observed RMS-derived condition stage, candidate samples are sorted by this source order. The ordered candidates are then partitioned into temporal segments according to the current per-stage memory quota. For K=100 and four observed stages at the final step, the quota is approximately 25 exemplars per stage; therefore each final-stage class is represented by about 25 temporal segments and 25 selected exemplars. For each segment, ASBR-CL computes the mean feature representation using the same MFF feature backbone used by the DGGN same-backbone baselines and ASBR-CL, and selects the candidate closest to that segment mean. This stage-aware memory strategy does not assume perfect monotonic separability of all degradation samples. It uses acquisition order as a practical proxy to preserve coverage across different degradation periods, so that the memory is not dominated by a local degradation interval and late or minority stages remain represented.

### 3.5. Balanced Replay

After the exemplar set is updated, the current training data and retained exemplars are combined for the next continual update. A standard shuffled loader can over-emphasize the current task when the current subset is much larger than the exemplar memory. ASBR-CL therefore uses the implemented balanced replay sampler when selected by the dataset configuration. For a mini-batch $B_{t}=B_{t}^{cur}\cup B_{t}^{mem}$, the old-sample replay ratio is

(2) $$\rho_{t}=\frac{|B_{t}^{mem}|}{|B_{t}^{cur}\left|+\right|B_{t}^{mem}|}.$$

The sampler assigns probability mass to current samples and old exemplars according to the configured $\rho_{t}$ and normalizes sample weights within each group. In the final configurations, SEU uses replay_old_ratio = 0.50, while XJTU uses replay_old_ratio = 0.30.

This mechanism is intentionally simple: it changes the sampling distribution used during incremental updates without introducing an additional loss term in the final selected method. The imbalance-aware baseline table checks whether balanced replay alone explains the XJTU result; the answer is negative because the balanced-replay baselines do not simultaneously match ASBR-CL-BoundedVal-K100 in Macro Recall and Average Forgetting.

### 3.6. Validation Checkpoint Selection

Validation checkpoint selection is treated as a conditional module. The implementation supports checkpoint strategies such as validation accuracy and validation Macro Recall, but ASBR-CL uses the module only when the validation-side evidence and the prespecified ablation protocol support it. When enabled, candidate checkpoints $\Theta_{t}$ from the update at step *t* are selected by validation Macro Recall,

(3) $$\theta_{t}^{⋆}=\arg\max_{\theta \in \Theta_{t}}\frac{1}{|Y_{1:t}|}\sum_{c\in Y_{1:t}}{\mathrm{Recall}}_{c}\left(\theta ;D_{1:t}^{\mathrm{val}}\right).$$

In the selected SEU configuration, checkpoint selection is disabled. In the selected XJTU configuration, the original manuscript used cumulative validation Macro Recall checkpoint selection, while the revision emphasizes ASBR-CL-BoundedVal-K100 as the more conservative setting with bounded validation samples.

The checkpoint module is therefore not an always-on component of ASBR-CL; it is a dataset-conditional model-selection step used when validation Macro Recall is aligned with the diagnostic objective. Test-set evidence is not used for epoch-level checkpoint selection.

### 3.7. Variant Selection Protocol

ASBR-CL contains optional modules: adaptive or stage-aware exemplar memory, balanced replay, and validation-based checkpoint selection. The final variant follows a predefined dataset-level protocol rather than post hoc selection from test-set performance. SEU is treated as a stable class-incremental gearbox fault diagnosis setting with balanced classes, where the main risk is old-class forgetting. Therefore, SEU uses random-boundary-diversity memory with a 0.10 random coverage ratio and balanced replay with old_ratio = 0.50, without validation checkpointing. XJTU-SY is treated as a degradation-stage incremental diagnosis setting with severe class/stage imbalance and adjacent RMS-derived stage confusion, where Macro Recall is a core objective. Therefore, XJTU-SY uses stage-aware memory, balanced replay with old_ratio = 0.30, and validation Macro Recall checkpoint selection when validation is allowed. The revision reports ASBR-CL-BoundedVal-K100 to make the validation resource explicit.

The test set was never used for epoch-level checkpoint selection or for selecting checkpoints. Test data were used only for final reporting after the dataset-level protocol and model variants had been fixed. The ablation results are reported as evidence for the predefined protocol rather than to define it post hoc.

### 3.8. Evaluation Metrics

The main metrics are Average Accuracy, Final Accuracy, Average Forgetting, Macro-F1, and Macro Recall. Let $A_{i,j}$ denote the accuracy on task or stage *j* after training has reached step *i*, with i≥j. Average Accuracy is the mean of the cumulative accuracies over the incremental steps, and Final Accuracy is the cumulative accuracy after the last step. Average Forgetting is computed as

(4) $$F=\frac{1}{T-1}\sum_{j=1}^{T-1}\left(\max_{i<T}A_{i,j}-A_{T,j}\right).$$

Signed backward transfer is reported as

(5) $$\mathrm{BWT}=\frac{1}{T-1}\sum_{j=1}^{T-1}\left(A_{T,j}-A_{j,j}\right).$$

Average Forgetting measures the performance drop from the best historical accuracy of each previous task or stage to the final model, whereas signed BWT measures the final change relative to the accuracy immediately after that task or stage was learned. Therefore, Average Forgetting and BWT are related retention metrics but are not generally equivalent. Higher values are better for the accuracy and balanced-recognition metrics, lower Average Forgetting indicates stronger retention, and a less negative BWT indicates less forgetting. Macro Recall is emphasized for degradation-stage diagnosis because it gives each class or stage equal weight and is less dominated by majority states than overall accuracy. This choice follows the broader evaluation principle that accuracy can be insufficient under class imbalance, where precision–recall and correlation-aware metrics provide complementary evidence [36,37].

## 4. Experiments

### 4.1. Datasets and Continual Learning Protocol

Experiments are conducted on two public diagnostic streams with complementary roles. Both datasets are treated as vibration sensor streams rather than static image-style classification tables: the continual protocol emulates an online diagnostic deployment in which new fault classes or degradation stages arrive after the model has already been trained on previous states. SEU-enhanced92 denotes the processed SEU gearbox stream used in this paper, constructed as the 92-dimensional feature construction for SEU from the Southeast University drivetrain dynamics simulator data as a five-class class-incremental stream. XJTU-bearing23 denotes the processed XJTU-SY bearing stream used in this paper, constructed as the 23-dimensional feature construction for XJTU-SY from the run-to-failure bearing data as a four-stage RMS-derived stage-incremental stream. SEU is linked to published SEU gearbox/bearing diagnosis resources [38,39], and XJTU follows the XJTU-SY accelerated life-test benchmark literature [40,41]. The selected ASBR-CL configuration follows the predefined dataset-level protocol in Section 3.7: SEU combines adaptive exemplar memory with balanced replay and no Macro Recall checkpointing; XJTU combines stage-aware memory, balanced replay, and validation Macro Recall checkpoint selection, with ASBR-CL-BoundedVal-K100 used as the conservative bounded-validation result in the revision. Historical training windows are not fully retained after each step; only the bounded exemplar memory is carried forward, matching memory-limited sensor data retention. All main accuracy and forgetting results are reported over five seeds: 42, 66, 2024, 3407, and 6546.

The feature construction is tabular and window based. For SEU-enhanced92, dimensions 1–81 are three channels times 27 time- and frequency-domain descriptors per channel: mean, standard deviation, root-mean-square (RMS), variance, median, quartiles, interquartile range, minimum, maximum, peak-to-peak amplitude, absolute mean, skewness, kurtosis, crest factor, shape factor, impulse factor, clearance factor, zero-crossing rate, line length, spectral centroid, spectral entropy, spectral flatness, dominant-frequency ratio, and low/mid/high band energies. Dimensions 82–92 are eleven global descriptors: three inter-channel correlations, global mean absolute value, global RMS, global maximum absolute value, global peak-to-peak amplitude, global energy, channel-RMS max/min ratio, channel-RMS entropy, and dominant-channel spectral entropy. Thus 3×27+11=92. For XJTU-bearing23, dimensions 1–5 are channel-0 mean, standard deviation, RMS, skewness, and kurtosis; dimensions 6–10 are the same five descriptors for channel 1; dimensions 11–15 reserve the same five descriptors for a third channel and are zero-filled because XJTU-SY provides only two vibration channels; and dimensions 16–23 are eight global descriptors: mean absolute value, maximum absolute value, peak-to-peak amplitude, energy, crest factor, shape factor, impulse factor, and dominant-channel spectral entropy. Thus 2×5+5+8=23. The zero-filled slots do not encode additional physical measurements; they keep the MFF/DGGN input layout fixed across the local feature pipeline. Table 1 makes the feature dimensions directly auditable.

Table 2 summarizes the dataset construction protocol. For SEU, the raw file headers indicate a DAQ frequency limit of 2000; the stream is therefore described as sampled at 2 kHz. The original files contain eight channels, and this study uses the three effective vibration channels from the planetary gearbox signals, corresponding to channels 2–4. Non-overlapping windows of length 2048 and stride 2048 are extracted. The final five classes are Health, Chipped, Miss, Root, and Surface under the 20-0 and 30-2 operating conditions. The split is performed within each source file by contiguous non-overlapping windows with a 60/20/20 train/validation/test ratio, giving 3060 training windows, 1020 validation windows, and 1030 test windows. For SEU, the split is segment-level with contiguous non-overlapping windows within each source file; therefore, it prevents direct window overlap but should not be interpreted as a source-file-level generalization split.

For XJTU-SY, the raw data contain 15 rolling bearings from accelerated run-to-failure tests under 35 Hz/12 kN, 37.5 Hz/11 kN, and 40 Hz/10 kN. The sampling frequency is 25.6 kHz. Each source record contains two vibration channels and 32,768 points, corresponding to 1.28 s, and is sampled once per minute. Each source record is split into eight non-overlapping windows of length 4096 and stride 4096. The final stage-incremental stream contains Normal, Slight, Moderate, and Severe stages. These stage labels are generated by the preprocessing procedure from a per-bearing RMS health indicator, not from test-set model performance. For each bearing run, source files are sorted by acquisition order. The procedure smooths per-file RMS with width 5, estimates baseline RMS as the median of the first 10% of the run, estimates failure RMS as the 95th percentile of the last 5%, computes $\mathrm{HI}=\mathrm{clip}(\left({\mathrm{RMS}}_{smooth}-\mathrm{baseline}\right)/\left(\mathrm{failure}-\mathrm{baseline}\right),0,1)$, and enforces monotonicity by a cumulative maximum over source order. Fixed thresholds then assign Normal for HI<0.20, Slight for 0.20≤HI<0.50, Moderate for 0.50≤HI<0.80, and Severe for 0.80≤HI≤1.00. XJTU uses a domain-stage stratified file-level split: source files are grouped by operating condition and generated degradation stage, assigned to train/validation/test at a 60/20/20 ratio, and all windows from the same source file remain in the same split. This prevents adjacent windows from the same sampled segment from leaking across train, validation, and test. The sample-level stage table and the stage-assignment procedure should be retained by the authors for audit, and sensitivity to alternative HI thresholds remains a separate robustness requirement.

All features use min-max normalization. The normalization parameters are fitted only on the training features and then applied to validation and test features. Validation and test data are not used to fit preprocessing statistics, which avoids preprocessing leakage. The validation split is used only for model selection and checkpoint selection. At step *t*, the validation set is cumulative over the observed classes or stages and contains both old-task and current-task validation data. It is not replay memory, is not used for training updates, and is not counted in the exemplar memory budget *K*. The test split is reserved for final result reporting. The feature list, preprocessing description, split construction, and XJTU stage-assignment rule above are reported to make the feature extraction and stage assignment auditable from the official raw datasets.

### 4.2. Baselines and Implementation Details

The revised memory-limited continual-learning comparisons focus on capacity-matched DGGN/MFF baselines. To address the capacity-mismatch concern, Fine-tuning, ER [33], iCaRL [32], and LwF [31] are re-run with the same DGGN/MFF backbone as ASBR-CL and are reported as DGGN-Fine-tuning, DGGN-ER, DGGN-iCaRL, and DGGN-LwF. The revised XJTU analysis also includes imbalance-aware same-backbone variants: DGGN-ER-BalancedReplay, DGGN-iCaRL-BalancedReplay, DGGN-ER-ClassBalancedLoss, DGGN-ER-FocalLoss, and DGGN-ER-BalancedSoftmax. These comparisons are treated as the primary reviewer-facing evidence. The DGGN with random-boundary memory row remains only as an implementation/timing reference from the original pipeline and is not used to support the revised capacity-matched claim. All revised memory-limited comparisons use the same incremental stream definition and fixed K=100 training-exemplar memory protocol.

### 4.3. Hyperparameter and Tuning Strategy

No new backbone or segmentation network is introduced in the revised method. The DGGN/MFF backbone and the basic optimizer settings follow the local DGGN-RBD implementation used for the same-backbone comparisons so that differences among ASBR-CL and the DGGN baselines are attributable to the continual-learning components rather than to a change in representation capacity. The fixed training settings are Adam, learning rate 0.1, weight decay $10^{-5}$, 30 epochs per incremental task, batch size 512 for SEU, and batch size 2048 for XJTU. The scheduler was retained for compatibility with the DGGN-RBD/local implementation but was not activated within the 30-epoch setting: the initialized MultiStepLR milestones at 200 and 400 are not reached, so the effective learning rate remains constant during each incremental task.

The continual-learning parameters are the training exemplar budget *K*, the replay ratio, the exemplar allocation rule, and the validation checkpoint strategy. The main experiments use K=100 retained training exemplars. The exemplar memory is allocated approximately uniformly over the currently observed classes or stages using $\left\lceil K/C_{t}\right\rceil$ exemplars when $C_{t}$ classes or stages have been observed; at the final step this gives about 20 exemplars per SEU class and 25 exemplars per XJTU stage. The selected old-sample replay ratio is 0.50 for SEU and 0.30 for XJTU. These continual-learning choices were fixed by the validation protocol or by the prespecified ablations reported in the revised evaluation. The test set was used only for final evaluation and was not used for hyperparameter tuning, variant selection, or checkpoint selection.

Table 3 summarizes the implementation details and tuning policy for the DGGN/MFF same-backbone and ASBR-CL runs.

### 4.4. Main Results

Table 4 reports the revised SEU same-backbone comparison. All rows use the DGGN/MFF backbone, five random seeds, and the same K=100 training-exemplar memory budget. Under this capacity-matched setting, ASBR-CL (selected SEU setting) obtains 97.49±1.18 Average Accuracy, 90.37±4.53 Final Accuracy, 90.19±4.65 Macro-F1, 90.37±4.53 Macro Recall, and 11.50±5.25 Average Forgetting. The same-backbone DGGN-ER baseline is the strongest SEU comparator in this revised table, but remains lower in balanced recognition and retention: 84.83±4.05 Macro-F1, 84.45±4.29 Macro Recall, and 18.20±5.15 Average Forgetting. These values support the SEU claim under a common DGGN/MFF representation rather than relying on smaller-capacity baselines.

Table 5 reports the revised XJTU same-backbone comparison. This table is the main capacity-mismatch response: DGGN-Fine-tuning, DGGN-ER, DGGN-iCaRL, DGGN-LwF, and ASBR-CL all use the DGGN/MFF backbone. The reviewer-compliant ASBR-CL-BoundedVal-K100 row uses K=100 retained training exemplars and caps the validation pool at 100 samples in total at each incremental update stage. This conservative row is the primary revised XJTU result. It is not uniformly stronger on every accuracy metric. In particular, DGGN-ER has slightly higher Final Accuracy than ASBR-CL-BoundedVal-K100 (89.52±2.68 versus 89.05±2.48). The ASBR-CL advantage is instead balanced recognition and retention: compared with DGGN-ER, ASBR-CL-BoundedVal-K100 improves Macro Recall from 63.94±7.50 to 75.00±2.00 and Macro-F1 from 55.48±12.82 to 67.66±3.83, while reducing Average Forgetting from 41.11±13.61 to 20.39±4.89. The original cumulative-validation ASBR-CL result remains in the table only as a reference to the submitted protocol.

Table 6 addresses the validation-resource concern. The original cumulative-validation setting used validation samples as a model-selection resource and did not count them in the training exemplar budget *K*. To make this resource explicit, the revision adds no-checkpoint, current-task-validation, and bounded-validation settings. ASBR-CL-BoundedVal-K100 caps the validation pool at 100 samples in total at each incremental update stage and remains close to the original cumulative-validation result: Average Accuracy changes from 94.51±0.80 to 94.06±1.12, Macro Recall from 75.88±2.39 to 75.00±2.00, and Macro-F1 from 68.69±2.75 to 67.66±3.83. The bounded result is therefore the more conservative XJTU version emphasized in the revised claim.

Table 7 addresses the XJTU stage-definition concern. The XJTU labels used here are RMS-derived condition-stage labels, not externally annotated degradation ground truth. They should therefore be interpreted as a reproducible condition-stage construction for continual diagnosis, and not as an absolute semantic annotation of true degradation states. As the RMS-derived granularity increases from three to five stages, the task becomes harder: Macro Recall decreases from 90.04±1.06 to 68.21±3.09, Macro-F1 decreases from 79.69±2.11 to 59.25±3.52, and Average Forgetting increases from 8.25±1.79 to 25.14±2.67.

Table 8 adds XJTU imbalance-aware same-backbone baselines. Balanced replay improves some baseline behavior: for example, DGGN-ER-BalancedReplay raises Average Accuracy from 87.20±8.58 to 92.90±1.85, and DGGN-iCaRL-BalancedReplay raises Average Accuracy from 66.34±10.41 to 90.52±3.29. However, these variants do not simultaneously match ASBR-CL-BoundedVal-K100 in Macro Recall and forgetting control. ASBR-CL-BoundedVal-K100 reaches 75.00±2.00 Macro Recall and 20.39±4.89 Average Forgetting, while the strongest balanced-replay comparator, DGGN-iCaRL-BalancedReplay, reaches 67.53±2.16 Macro Recall and 34.54±4.29 Average Forgetting. This supports interpreting ASBR-CL as the combined effect of stage-aware exemplar memory, balanced replay, and validation checkpointing, not as balanced replay alone.

### 4.5. Statistical Significance

Table 9 summarizes the reviewer-relevant XJTU paired tests computed from the five-seed experimental results. Comparisons are paired by the five random seeds and use ASBR-CL-BoundedVal-K100 as the revised ASBR-CL reference. The table reports paired *t*-tests, Wilcoxon signed-rank tests, Holm-adjusted *p*-values, and Cohen’s $d_{z}$. Because n=5 is small and multiple metrics are tested, the statistical evidence is interpreted cautiously. The clearest Holm-adjusted result is the Macro-F1 improvement over DGGN-iCaRL-BalancedReplay ($p_{\mathrm{Holm}}=0.01076$, $d_{z}=4.740$). The Macro Recall comparison against DGGN-iCaRL-BalancedReplay is close to but not below the conventional 0.05 threshold after Holm adjustment ($p_{\mathrm{Holm}}=0.05028$). Other balanced-recognition and forgetting differences have favorable effect-size directions but do not remain significant after Holm adjustment, so the manuscript does not claim that every comparison is statistically significant.

**Figure 3:**
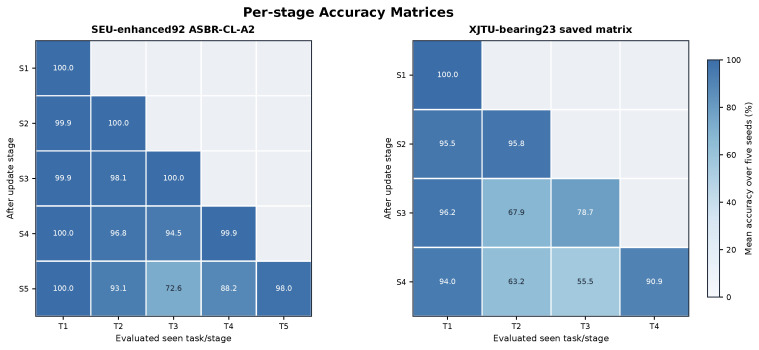
Per-stage accuracy-matrix heatmaps for the continual diagnostic streams. Rows are training stages after each incremental update, columns are evaluated seen tasks or RMS-derived degradation stages, and values are mean accuracies over five seeds computed from saved task accuracy matrices.

**Figure 4:**
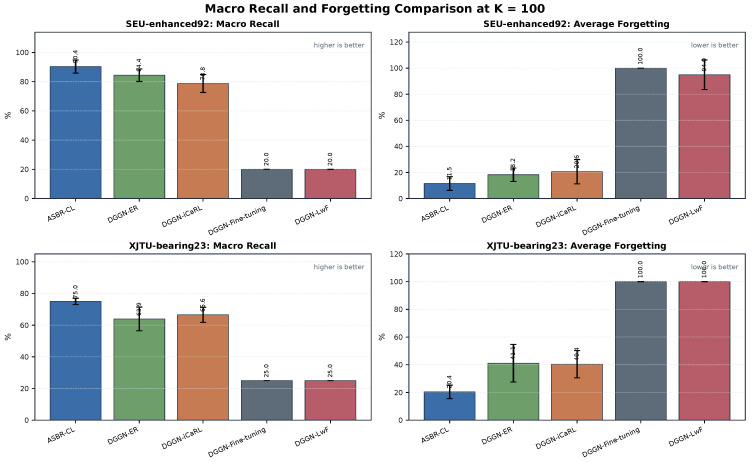
Final-stage Macro Recall and Average Forgetting comparison under the memory-limited continual-learning protocol with K=100. Bars and error bars report mean ± standard deviation from the saved five-seed result tables. Higher Macro Recall is better and lower Average Forgetting is better.

**Figure 5:**
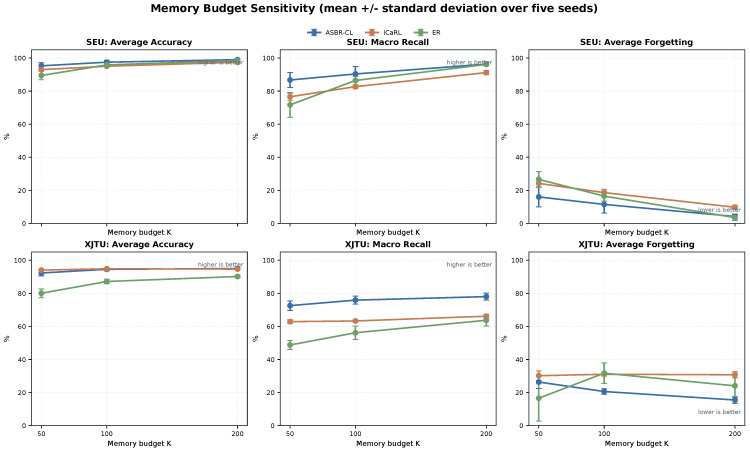
Average-over-stage memory-budget sensitivity comparison under K=50, K=100, and K=200. The budget *K* counts only retained training exemplars in replay memory, and plotted values are mean ± standard deviation over five seeds recomputed from saved result files.

Figure 3 complements the aggregate tables by showing the per-stage accuracy matrices, while Figure 4 emphasizes final-stage Macro Recall and Average Forgetting. Figure 5 places the selected K=100 setting in the average-over-stage memory-budget sensitivity context.

**Figure 6:**
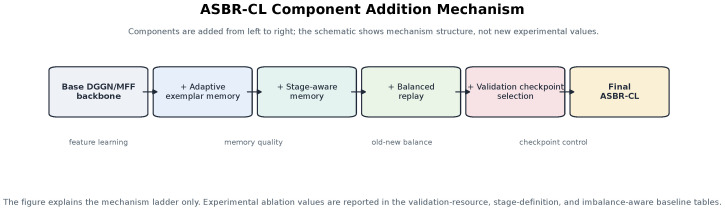
Component-addition mechanism summary for the ASBR-CL ablation logic. The ladder starts from the base DGGN/MFF backbone and adds adaptive exemplar memory, stage-aware memory, balanced replay, and validation checkpoint selection before the final ASBR-CL configuration. The figure is a mechanism schematic; the corresponding numerical ablation evidence is reported in the validation-resource, stage-definition, and imbalance-aware baseline tables.

Figure 6 provides a mechanism-level summary of the ablation logic before the numerical ablation evidence is discussed.

### 4.6. Ablation Study

The revised ablation evidence is reported through three targeted tables rather than by relying on a single component ladder. First, Table 6 isolates checkpoint and validation-resource effects on XJTU. Removing checkpoint selection reduces Average Accuracy to 91.69±2.19 and increases Average Forgetting to 27.49±3.49, whereas the bounded-validation setting reaches 94.06±1.12 Average Accuracy and 20.39±4.89 Average Forgetting. Second, Table 7 shows that the RMS-derived stage granularity materially changes the difficulty of the task; Macro Recall falls from 90.04±1.06 for three stages to 68.21±3.09 for five stages. Third, Table 8 separates balanced-replay and imbalance-aware losses from the full ASBR-CL mechanism. Balanced replay improves some baseline Average Accuracy values, but it does not reproduce the ASBR-CL-BoundedVal-K100 combination of 75.00±2.00 Macro Recall and 20.39±4.89 Average Forgetting. These ablations support the interpretation that the XJTU result comes from the combination of stage-aware exemplar memory, balanced replay, and validation checkpointing under a bounded validation resource, not from balanced replay alone.

### 4.7. Memory Budget Sensitivity

Figure 5 compares ASBR-CL, iCaRL, and ER under K=50, K=100, and K=200. The selected K=100 setting is therefore reported together with neighboring memory budgets to show whether the conclusion depends on a single exemplar capacity.

### 4.8. Cost Analysis

**Figure 7:**
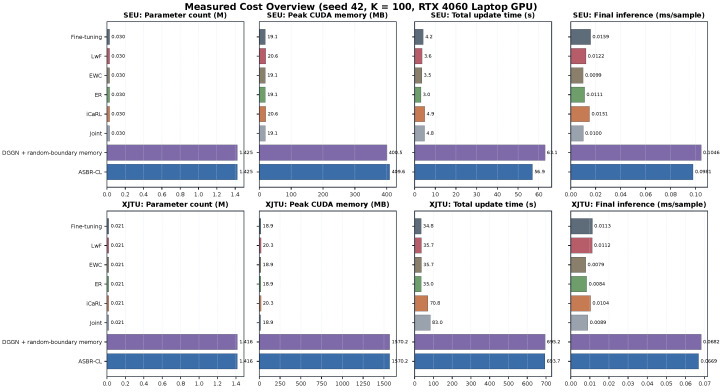
Measured cost overview under seed 42, K=100, and an RTX 4060 Laptop GPU. Parameter count, peak GPU memory, total training/update time, and final inference time are parsed from the timing logs. Update time is the total wall-clock update cost accumulated over the continual stages, and inference time is reported as milliseconds per sample at final evaluation.

**Figure 8:**
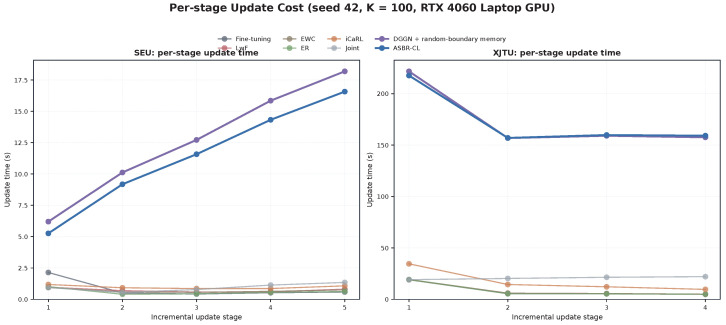
Per-stage update cost under seed 42, K=100, and an RTX 4060 Laptop GPU. Values are parsed directly from the stage-update-time arrays in the timing logs and report the wall-clock update time for each incremental stage rather than a total or multi-seed average.

Cost is evaluated with the same memory-limited continual-learning setting used in the main experiments: seed 42, memory budget K=100, and an RTX 4060 Laptop GPU. Figure 7 summarizes the measured parameter count, peak GPU memory, total update time, and final inference time, while Figure 8 shows the per-stage update cost parsed from the timing logs. The numeric values in Table 10 are taken from the real timing table and timing logs rather than being inferred from the figures. Because the timing is measured for a single seed and one hardware configuration, it should be interpreted as an exploratory implementation-cost measurement rather than a stable multi-run cost estimate. The reported update time is the total wall-clock time accumulated over continual update stages in the implemented training/evaluation loop for seed 42. It includes the validation checkpoint evaluation executed when the selected protocol enables it, so the XJTU validation-assisted timing includes the implemented cumulative-validation checkpoint-selection pass. It does not separately decompose offline feature extraction, pre-generated data loading, training time, validation checkpointing time, model-selection overhead, final evaluation time, or multi-seed timing variability.

The cost results show that ASBR-CL is not the cheapest method in raw update time or memory footprint. Its DGGN backbone and replay state make it substantially heavier than Fine-tuning, LwF, EWC, ER, and iCaRL in peak GPU memory. This cost must be interpreted together with the diagnostic objective: ASBR-CL is designed to improve balanced recognition and retention under a bounded exemplar memory, not to minimize update time alone.

Compared with the DGGN + random-boundary memory timing reference, however, ASBR-CL stays in the same update-time order. On SEU, ASBR-CL requires 56.92 s versus 63.08 s for that reference; on XJTU, it requires 693.68 s versus 695.21 s. The adaptive memory, balanced replay, and conditional checkpoint behavior therefore do not change the method into a low-cost baseline, but they also do not introduce a separate order of computational cost relative to the DGGN/MFF implementation reference. Joint Training is reported only as an offline upper bound because it has access to the full historical training set and is not a memory-limited continual-learning method.

## 5. Discussion

The two datasets support different result patterns. SEU provides the cleaner same-backbone result: the selected ASBR-CL setting improves the reported capacity-matched metrics over DGGN-ER, DGGN-iCaRL, DGGN-LwF, and DGGN-Fine-tuning under the selected protocol. The revised SEU evidence should be interpreted through the same DGGN/MFF backbone comparison rather than through smaller-capacity baselines.

XJTU is more informative as a hard-case study. ASBR-CL-BoundedVal-K100 is not universally superior on all accuracy metrics, especially Final Accuracy. DGGN-ER reaches a slightly higher Final Accuracy (89.52±2.68 versus 89.05±2.48), so the revised XJTU claim is not an accuracy-only claim. The ASBR-CL advantage lies instead in balanced recognition and retention: it reports 75.00±2.00 Macro Recall, 67.66±3.83 Macro-F1, and 20.39±4.89 Average Forgetting, compared with DGGN-ER at 63.94±7.50, 55.48±12.82, and 41.11±13.61. This distinction matters for safety-critical diagnosis: imbalanced diagnostic and evaluation studies show that conventional accuracy can be misleading when minority or out-of-distribution states are safety-relevant [11,28,36,42,43]. Macro Recall gives each class or stage equal weight, and Average Forgetting reflects how much previously learned diagnostic behavior is lost after later updates.

The XJTU labels should also be interpreted carefully. The Normal, Slight, Moderate, and Severe labels in XJTU-bearing23 are RMS-derived condition stages, not externally annotated degradation ground truth. Table 7 therefore reports a sensitivity analysis across 3-stage, 4-stage, and 5-stage RMS-derived definitions. The trend is expected: increasing stage granularity makes the task harder and reduces Macro Recall and Macro-F1. This sensitivity analysis supports the use of the labels as reproducible condition stages while limiting claims about absolute degradation-state semantics.

The cost analysis also narrows the interpretation. ASBR-CL is not a low-cost method compared with the smaller feature-level continual-learning reference baselines. Its update time and peak memory are close to the DGGN/MFF implementation reference and much larger than those of Fine-tuning, LwF, EWC, ER, and iCaRL. The practical argument is therefore not that ASBR-CL minimizes cost, but that it obtains stronger balanced recognition and retention without moving beyond the update-time order of the DGGN/MFF reference implementation. Whether this trade-off is acceptable depends on the deployment setting. For applications where update latency is the dominant constraint, simpler replay methods may be preferable. For safety-critical degradation diagnosis, balanced recognition and reduced forgetting may justify the additional cost.

### Practical Implications

ASBR-CL is most suitable when a diagnostic model is updated incrementally, historical data cannot be fully retained, and minority or late degradation stages must remain recognizable. Simpler ER or iCaRL variants may be more appropriate when update latency, memory footprint, or implementation simplicity is the dominant deployment requirement and overall accuracy is the main operating metric. When the application is safety-critical and missed recognition of minority degradation stages is costly, the Macro Recall and forgetting gains of ASBR-CL provide a stronger practical motivation, provided that the DGGN-level update cost is acceptable.

The current study has limitations. First, the evidence is based on two public datasets and a fixed memory-budget protocol centered on K=100, although memory sensitivity is also visualized. Second, the dataset splits have different generalization meanings: for SEU, the split is segment-level with contiguous non-overlapping windows within each source file and should not be interpreted as a source-file-level generalization split; for XJTU, all windows from the same source file remain in the same split. Third, XJTU stage labels are RMS-derived condition labels rather than externally annotated degradation ground truth. The 3/4/5-stage sensitivity table reduces this risk but does not replace external health-state annotation. Fourth, validation memory in the original cumulative setting is a model-selection resource and separate from training exemplar memory; the new ASBR-CL-BoundedVal-K100 row is more conservative, but stricter streaming validation protocols remain future work. Fifth, ASBR-CL is not universally superior on all accuracy metrics, especially XJTU Final Accuracy. Sixth, same-backbone and imbalance-aware baselines have now been added to reduce capacity and imbalance confounds, but broader industrial datasets, unseen-equipment splits, external health-state annotations, and stricter streaming validation should be explored in future work. The cost table is measured for seed 42 only and does not decompose feature extraction, data loading, training, validation checkpointing, model selection, and final evaluation into separate columns; multi-seed cost means and standard deviations are therefore not claimed. Finally, the statistical evidence is based on five seeds. The revised statistical table reports paired t-tests, Wilcoxon signed-rank tests, Holm-adjusted *p*-values, and Cohen’s $d_{z}$, but because n=5 is small, significance conclusions should remain cautious.

Another limitation is that the present experiments focus on single-fault or single-condition-stage continual diagnosis. The SEU setting is constructed as a class-incremental single-fault stream, and the XJTU-SY setting is constructed as an RMS-derived degradation-stage stream. Compound faults may introduce interacting fault signatures, multi-label outputs, and more severe overlap between classes or stages. The exemplar-memory, balanced-replay, and conditional checkpoint-selection mechanisms in ASBR-CL are in principle compatible with such a setting, because they are designed to control old-new imbalance and forgetting under bounded memory when new diagnostic states arrive. However, the present experiments do not directly validate compound-fault diagnosis, and we therefore do not claim validated compound-fault performance. Extending ASBR-CL to compound-fault and multi-label continual diagnosis is left as future work.

## 6. Conclusions

This paper presented ASBR-CL, a continual fault diagnosis framework under a fixed training-exemplar memory budget that combines adaptive/stage-aware exemplar memory, balanced replay, and conditional validation checkpoint selection. The method is dataset-aware under a predefined variant selection protocol: SEU uses adaptive exemplar memory and balanced replay without validation checkpointing, while XJTU uses stage-aware memory, balanced replay, and validation Macro Recall checkpoint selection, with ASBR-CL-BoundedVal-K100 reported as the conservative bounded-validation setting.

The experimental evidence supports a bounded but positive conclusion. On SEU, ASBR-CL improves the reported same-backbone metrics under the selected no-validation-checkpoint protocol. On XJTU, ASBR-CL-BoundedVal-K100 improves Macro Recall, Macro-F1, and forgetting control under a fixed exemplar-memory budget and bounded validation setting, but it is not a universal accuracy winner; DGGN-ER has slightly higher Final Accuracy. Real timing measurements further show that ASBR-CL is not the cheapest method, but remains in the same update-time order as the DGGN/MFF implementation reference while providing stronger balanced recognition and retention.

Overall, ASBR-CL improves balanced recognition and forgetting control under continual fault diagnosis with a fixed training-exemplar memory budget, while making cost and validation-resource trade-offs explicit. The results suggest that continual fault diagnosis should be evaluated by accuracy, balanced recognition, forgetting, training-memory use, validation-resource use, test-resource separation, and update cost together. Future work should extend this evaluation to external health-state annotations, more industrial machinery datasets, broader memory budgets, stricter streaming validation protocols, and validation-free or weakly supervised checkpoint selection for deployment settings where labeled validation data are scarce.

## Figures and Tables

**Table 1:** Feature-dimension accounting for the processed tabular inputs.

Dataset	Dimension Range	Source	Descriptors/Meaning
SEU-enhanced92	1–81	three real vibration channels	27 time/frequency descriptors per channel
SEU-enhanced92	82–92	global features	inter-channel correlation, global amplitude/energy, channel-RMS ratio/entropy, dominant-channel entropy
XJTU-bearing23	1–5	horizontal channel	mean, standard deviation, RMS, skewness, kurtosis
XJTU-bearing23	6–10	vertical channel	mean, standard deviation, RMS, skewness, kurtosis
XJTU-bearing23	11–15	zero-filled alignment slots	reserved third-channel descriptors; no physical third-channel measurement in XJTU-SY
XJTU-bearing23	16–23	global features	mean absolute value, maximum absolute value, peak-to-peak amplitude, energy, crest/shape/impulse factors, dominant-channel entropy

**Table 2:** Dataset construction and continual-learning protocol. Splits are reported as train/validation/test counts.

Dataset	Source and Channels	Sampling/Window	Split and Leakage Control	Continual Tasks	Task Counts
SEU	SEU drivetrain; 3 vibration channels, ch. 2–4; domains 20-0 and 30-2	2 kHz; length 2048; stride 2048; non-overlapping	60/20/20 contiguous split within each source file; no overlapping cross-split windows	Class-incremental; T1 Health + Chipped; T2 Miss; T3 Root; T4 Surface	Total 3060/1020/1030; T1 1224/408/412; T2–T4 each 612/204/206
XJTU-bearing23	XJTU-SY run-to-failure bearings; 2 vibration channels; 35 Hz/12 kN, 37.5 Hz/11 kN, 40 Hz/10 kN	25.6 kHz; source record 32,768 points; 8 windows per record; length 4096; stride 4096	60/20/20 domain-stage stratified file-level split; one source record remains in one split	Stage-incremental; T1 Normal; T2 Slight; T3 Moderate; T4 Severe	Total 44,240/14,760/14,728; Normal 37,608/12,544/12,520; Slight 3448/1152/1144; Moderate 2368/792/792; Severe 816/272/272

**Table 3:** Implementation details and tuning policy for the DGGN/MFF same-backbone and ASBR-CL runs over five seeds. All rows use K=100 retained training exemplars in the main memory-limited comparisons; validation data are model-selection resources and test data are reserved for final evaluation.

Setting	SEU-enhanced92	XJTU-bearing23
Backbone	MFF feature backbone	MFF feature backbone
Optimizer	Adam	Adam
Learning rate/weight decay	0.1/$10^{-5}$	0.1/$10^{-5}$
Epochs per incremental task	30	30
Scheduler	MultiStepLR milestones 200, 400; γ=0.2; not triggered within 30 epochs	MultiStepLR milestones 200, 400; γ=0.2; not triggered within 30 epochs
Batch size	512	2048
Random seeds	42, 66, 2024, 3407, 6546	42, 66, 2024, 3407, 6546
Default memory budget	K=100 training exemplars; validation excluded	K=100 training exemplars; validation excluded
Memory allocation	Uniform over observed classes; about 20 exemplars/class at final step	Uniform over observed stages; about 25 exemplars/stage at final step
Final ASBR-CL setting	Selected SEU setting: adaptive exemplar memory + balanced replay, no validation checkpoint	ASBR-CL-BoundedVal-K100: stage-aware memory + balanced replay + bounded validation Macro Recall checkpoint
Old-sample replay ratio	0.50	0.30

**Table 4:** SEU-enhanced92 same-backbone results over five seeds. Values are mean ± standard deviation in percent. All methods use the DGGN/MFF backbone and K=100 retained training exemplars; validation samples are model-selection resources and are not counted in *K*. Signed BWT is recomputed from the saved stage accuracy matrices using the definition in Section 3.8.

Method	Avg. Acc.	Final Acc.	Avg. Forgetting	Signed BWT	Macro-F1	Macro Recall
ASBR-CL (selected SEU)	97.49±1.18	90.37±4.53	11.50±5.25	−11.50±5.25	90.19±4.65	90.37±4.53
DGGN-Fine-tuning	45.67±0.00	20.00±0.00	100.00±0.00	−100.00±0.00	6.67±0.00	20.00±0.00
DGGN-ER	94.64±1.64	84.45±4.29	18.20±5.15	−18.20±5.15	84.83±4.05	84.45±4.29
DGGN-iCaRL	93.55±1.71	78.76±6.16	20.58±9.31	−20.58±9.31	77.56±7.80	78.76±6.16
DGGN-LwF	45.70±0.08	20.02±0.04	94.93±11.34	−94.93±11.34	6.71±0.09	20.02±0.04

**Table 5:** XJTU-bearing23 same-backbone results over five seeds. Values are mean ± standard deviation in percent. All rows use the DGGN/MFF backbone and K=100 retained training exemplars. ASBR-CL-BoundedVal-K100 is the conservative bounded-validation result used for the revised XJTU claim; ASBR-CL-CumulativeVal-original is shown only as the original manuscript protocol. Signed BWT is recomputed from the saved stage accuracy matrices using the definition in Section 3.8.

Method	Avg. Acc.	Final Acc.	Avg. Forgetting	Signed BWT	Macro-F1	Macro Recall
ASBR-CL-BoundedVal-K100	94.06±1.12	89.05±2.48	20.39±4.89	−20.39±4.89	67.66±3.83	75.00±2.00
ASBR-CL-CumulativeVal-original	94.51±0.80	89.49±1.49	20.63±1.70	−20.63±1.70	68.69±2.75	75.88±2.39
DGGN-Fine-tuning	28.92±0.00	1.85±0.00	100.00±0.00	−100.00±0.00	0.91±0.00	25.00±0.00
DGGN-ER	87.20±8.58	89.52±2.68	41.11±13.61	−41.11±13.61	55.48±12.82	63.94±7.50
DGGN-iCaRL	66.34±10.41	83.44±5.11	40.38±9.85	−40.38±9.85	51.30±5.53	66.55±4.75
DGGN-LwF	28.92±0.00	1.85±0.00	100.00±0.00	−100.00±0.00	0.91±0.00	25.00±0.00

**Table 6:** XJTU validation-resource ablation over five seeds with DGGN/MFF same-backbone ASBR-CL and K=100 retained training exemplars. Validation samples are used only for model selection and checkpoint selection, not for replay training memory. ASBR-CL-BoundedVal-K100 keeps at most 100 validation samples in total for checkpoint selection at each update stage and is the conservative reviewer-compliant XJTU row.

Variant	Validation Pool by Update Stage	Avg. Acc.	Final Acc.	Avg. Forgetting	Signed BWT	Macro-F1	Macro Recall
ASBR-CL-CumulativeVal-original	12,544/13,696/14,488/14,760	94.51±0.80	89.49±1.49	20.63±1.70	−20.63±1.70	68.69±2.75	75.88±2.39
ASBR-CL-NoCkpt	0/0/0/0	91.69±2.19	86.59±2.49	27.49±3.49	−27.49±3.49	62.68±3.05	71.84±2.91
ASBR-CL-CurrentVal	12,544/1152/792/272	93.20±1.17	87.76±3.43	20.99±4.05	−20.99±4.05	65.03±3.14	71.81±1.25
ASBR-CL-BoundedVal-K100	100/100/100/100	94.06±1.12	89.05±2.48	20.39±4.89	−20.39±4.89	67.66±3.83	75.00±2.00

**Table 7:** XJTU stage-definition sensitivity over five seeds. Labels are RMS-derived condition stages rather than external human-annotated degradation ground truth. All rows use ASBR-CL with the DGGN/MFF backbone, K=100 retained training exemplars, and bounded validation samples matched to the number of stages.

Stage Definition	Avg. Acc.	Final Acc.	Avg. Forgetting	Signed BWT	Macro-F1	Macro Recall
3-stage RMS-derived labels	96.54±0.57	93.40±0.89	8.25±1.79	−8.25±1.79	79.69±2.11	90.04±1.06
4-stage RMS-derived labels	94.06±1.12	89.05±2.48	20.39±4.89	−20.39±4.89	67.66±3.83	75.00±2.00
5-stage RMS-derived labels	91.08±2.25	84.21±3.42	25.14±2.67	−25.14±2.67	59.25±3.52	68.21±3.09

**Table 8:** XJTU imbalance-aware same-backbone baselines over five seeds. All methods use the DGGN/MFF backbone and K=100 retained training exemplars. ASBR-CL-BoundedVal-K100 uses bounded validation and is included as the conservative revised ASBR-CL reference.

Method	Avg. Acc.	Final Acc.	Avg. Forgetting	Signed BWT	Macro-F1	Macro Recall
DGGN-ER	87.20±8.58	89.52±2.68	41.11±13.61	−41.11±13.61	55.48±12.82	63.94±7.50
DGGN-iCaRL	66.34±10.41	83.44±5.11	40.38±9.85	−40.38±9.85	51.30±5.53	66.55±4.75
DGGN-ER-BalancedReplay	92.90±1.85	89.29±2.02	42.38±12.61	−42.38±12.61	50.17±10.11	63.54±7.26
DGGN-iCaRL-BalancedReplay	90.52±3.29	88.03±1.61	34.54±4.29	−34.54±4.29	56.18±2.99	67.53±2.16
DGGN-ER-ClassBalancedLoss	89.41±8.47	71.05±36.49	34.22±12.06	−34.22±12.06	50.26±19.86	61.26±14.75
DGGN-ER-FocalLoss	86.20±7.90	60.07±34.90	46.77±18.36	−46.73±18.41	42.22±17.69	52.61±9.09
DGGN-ER-BalancedSoftmax	91.02±2.95	79.21±12.22	44.33±16.42	−44.33±16.42	43.03±12.38	57.60±7.46
ASBR-CL-BoundedVal-K100	94.06±1.12	89.05±2.48	20.39±4.89	−20.39±4.89	67.66±3.83	75.00±2.00

**Table 9:** Reviewer-relevant XJTU paired statistical tests over five seeds. ASBR-CL refers to ASBR-CL-BoundedVal-K100. Diff. is ASBR-CL minus baseline in percentage points; for Average Forgetting, negative Diff. means lower forgetting for ASBR-CL. Holm-adjusted *p*-values for the paired *t*-tests and Wilcoxon signed-rank tests are computed from the five paired seed-level results.

Baseline	Metric	Diff. pp	Paired *t*-Test *p*	$p_{\mathrm{Holm}}$	Wilcoxon *p*	Wilcoxon $p_{\mathrm{Holm}}$	Cohen’s $d_{z}$
DGGN-ER	Final Accuracy	−0.48	0.4201	1.0000	0.4375	1.0000	−0.401
DGGN-ER	Avg. Forgetting	−20.72	0.04644	0.5780	0.1250	1.0000	−1.274
DGGN-ER	Macro-F1	+12.18	0.1138	0.7966	0.0625	1.0000	0.902
DGGN-ER	Macro Recall	+11.06	0.04782	0.5780	0.0625	1.0000	1.261
DGGN-iCaRL	Final Accuracy	+5.61	0.04334	0.5780	0.0625	1.0000	1.305
DGGN-iCaRL	Avg. Forgetting	−19.99	0.03392	0.5780	0.1250	1.0000	−1.417
DGGN-iCaRL	Macro-F1	+16.36	0.01226	0.2330	0.0625	1.0000	1.940
DGGN-iCaRL	Macro Recall	+8.45	0.04089	0.5780	0.1250	1.0000	1.331
DGGN-ER-BalancedReplay	Final Accuracy	−0.24	0.9069	1.0000	1.0000	1.0000	−0.056
DGGN-ER-BalancedReplay	Avg. Forgetting	−21.99	0.03554	0.5780	0.0625	1.0000	−1.395
DGGN-ER-BalancedReplay	Macro-F1	+17.49	0.03211	0.5780	0.0625	1.0000	1.443
DGGN-ER-BalancedReplay	Macro Recall	+11.46	0.03326	0.5780	0.0625	1.0000	1.426
DGGN-iCaRL-BalancedReplay	Final Accuracy	+1.01	0.5822	1.0000	0.8125	1.0000	0.267
DGGN-iCaRL-BalancedReplay	Avg. Forgetting	−14.15	0.006369	0.1338	0.0625	1.0000	−2.340
DGGN-iCaRL-BalancedReplay	Macro-F1	+11.48	0.0004483	0.01076	0.0625	1.0000	4.740
DGGN-iCaRL-BalancedReplay	Macro Recall	+7.47	0.002186	0.05028	0.0625	1.0000	3.133

**Table 10:** Core real update-cost indicators measured with seed 42, K=100, and an RTX 4060 Laptop GPU. The table is split by dataset for readability. Joint Training is included only as an offline upper bound, not as a same-cost continual-learning baseline. DGGN + random-boundary memory is retained as the original implementation/timing reference, while the reviewer-facing capacity-matched accuracy comparisons are reported in Table 4, Table 5 and Table 8.

**SEU-enhanced92**					
**Method**	**Setting**	**Params (M)**	**Peak GPU (MB)**	**Update (s)**	**Infer. (ms/sample)**
Fine-tuning	memory-limited CL	0.030	19.1	4.18	0.0159
LwF	memory-limited CL	0.030	20.6	3.64	0.0122
EWC	memory-limited CL	0.030	19.1	3.48	0.0099
ER	memory-limited CL	0.030	19.1	3.04	0.0111
iCaRL	memory-limited CL	0.030	20.6	4.90	0.0151
Joint	offline upper bound	0.030	19.1	4.76	0.0100
DGGN + random-boundary memory	memory-limited CL	1.425	400.5	63.08	0.1046
ASBR-CL	memory-limited CL	1.425	409.6	56.92	0.0981
**XJTU-bearing23**					
**Method**	**Setting**	**Params (M)**	**Peak GPU (MB)**	**Update (s)**	**Infer. (ms/sample)**
Fine-tuning	memory-limited CL	0.021	18.9	34.84	0.0113
LwF	memory-limited CL	0.021	20.3	35.65	0.0112
EWC	memory-limited CL	0.021	18.9	35.71	0.0079
ER	memory-limited CL	0.021	18.9	34.98	0.0084
iCaRL	memory-limited CL	0.021	20.3	70.82	0.0104
Joint	offline upper bound	0.021	18.9	82.97	0.0089
DGGN + random-boundary memory	memory-limited CL	1.416	1570.2	695.21	0.0682
ASBR-CL	memory-limited CL	1.416	1570.2	693.68	0.0669

## Data Availability

The data that support the findings of this study are openly available in the following repositories: SEU Mechanical Dataset: https://github.com/cathysiyu/Mechanical-datasets (accessed on 7 June 2026); XJTU-SY Bearing Dataset: https://biaowang.tech/xjtu-sy-bearing-datasets/ (accessed on 7 June 2026).
